# The Association of Virulence Factors with Genomic Islands

**DOI:** 10.1371/journal.pone.0008094

**Published:** 2009-12-01

**Authors:** Shannan J. Ho Sui, Amber Fedynak, William W. L. Hsiao, Morgan G. I. Langille, Fiona S. L. Brinkman

**Affiliations:** Department of Molecular Biology and Biochemistry, Simon Fraser University, Burnaby, British Columbia, Canada; University of Hyderabad, India

## Abstract

**Background:**

It has been noted that many bacterial virulence factor genes are located within genomic islands (GIs; clusters of genes in a prokaryotic genome of probable horizontal origin). However, such studies have been limited to single genera or isolated observations. We have performed the first large-scale analysis of multiple diverse pathogens to examine this association. We additionally identified genes found predominantly in pathogens, but not non-pathogens, across multiple genera using 631 complete bacterial genomes, and we identified common trends in virulence for genes in GIs. Furthermore, we examined the relationship between GIs and clustered regularly interspaced palindromic repeats (CRISPRs) proposed to confer resistance to phage.

**Methodology/Principal Findings:**

We show quantitatively that GIs disproportionately contain more virulence factors than the rest of a given genome (p<1E-40 using three GI datasets) and that CRISPRs are also over-represented in GIs. Virulence factors in GIs and pathogen-associated virulence factors are enriched for proteins having more “offensive” functions, e.g. active invasion of the host, and are disproportionately components of type III/IV secretion systems or toxins. Numerous hypothetical pathogen-associated genes were identified, meriting further study.

**Conclusions/Significance:**

This is the first systematic analysis across diverse genera indicating that virulence factors are disproportionately associated with GIs. “Offensive” virulence factors, as opposed to host-interaction factors, may more often be a recently acquired trait (on an evolutionary time scale detected by GI analysis). Newly identified pathogen-associated genes warrant further study. We discuss the implications of these results, which cement the significant role of GIs in the evolution of many pathogens.

## Introduction

The establishment of infection is mediated by virulence factors, which can be generally defined as bacterial products or strategies that contribute to the ability of the bacterium to cause disease. Most bacterial virulence factors were originally thought to be associated with pathogens. However, as genome sequences from non-pathogenic, commensal bacteria were obtained, it became clear that many “classic” virulence factors, such as adhesions, were also encoded in the genomes of commensal bacteria [1], [2]. It has therefore been proposed that such virulence factors should be more generally referred to as “host-interaction factors” [3]. Microarray analyses also supported these findings; for example, many of the known virulence associated genes in pathogenic *Neisseria* sp., were also found to be present in the closely-related non-pathogen *Neisseria lactamica* [4]. However, it is evident that certain types of genes, such as botulinum toxin, are both necessary and sufficient to cause disease on their own [5]. While it is generally appreciated that microbial pathogenesis is a complex process that reflects an interplay of pathogen, host, and environmental factors, we wished to examine to what degree there may be virulence factors that are so critical for disease processes that their very presence is strongly associated with disease, rather than simply host colonization/interaction.

With the number and diversity of bacterial genomes sequenced, we can investigate selected observations regarding pathogenicity and quantify them on a more global scale. In particular, it has been noted that many virulence genes are associated with genomic islands (GIs; clusters of genes of probable horizontal origin) [6]–[13]. The first GIs identified were in fact called pathogenicity islands (PAIs) [2], [14]. Since then, many others have frequently noted the apparent association of virulence factors with such horizontally acquired regions (reviewed in [2], [7], [8], [10]–[13], [15], [16]). However, no analysis has yet been reported that examines whether this trend is systematically true across diverse lineages of pathogens.

Such an analysis is now possible as methods for high quality GI prediction have been developed and we have access to additional datasets of known GIs [17], [18]. In addition, a curated dataset of virulence factors is available through the Virulence Factor Database (VFDB) [19], [20], which may be cross-referenced with current bacterial genome datasets. The numerous genomes that have been sequenced from both pathogenic and non-pathogenic strains of diverse bacterial genera permit us to investigate the degree to which there are classes of genes that may be pathogen-specific or notably pathogen-associated. Previous analyses of pathogen-specific genes have been limited to certain species or genera (for example, [4], [21]–[25]), but a large-scale analysis is now possible. While such an analysis is still limited by the scope of bacterial genome sequences and virulence factors currently available, any virulence factors observed to be present in pathogenic strains from diverse bacterial genera, with no detectable homologs in non-pathogenic strains of the same genera, are considered good candidates for being classified as pathogen-associated. We set out to examine whether such genes could be identified within a diverse bacterial genome dataset, and to examine common features of such genes with the hypothesis that they may play more virulence-specific roles in pathogens. Such genes also represent targets for possible novel therapeutic strategies that interfere with pathogen-specific traits that contribute to pathogenesis [26], [27].

For this study, we characterized the prevalence of pathogen-associated virulence factors, and virulence factors in general, in both whole bacterial genomes and in GIs. We show that certain types of virulence factors are strongly associated with both pathogens and GIs. We note that our definition of a virulence factor simply requires that the gene be known to be involved in virulence in one host to date. Although any given virulence factor may be essential to pathogenesis in some hosts but not others, this simple definition allows us to examine all genes found to be involved in virulence and compare them to genes that have not been found to be involved in virulence in any host to date, providing insights into general trends that is not possible through targeted analysis of individual pathogens or genes. The implications of our results on therapeutic development and the evolution of pathogenicity are discussed.

## Results

### Virulence factors are disproportionately found in genomic islands

Isolated studies of selected, closely related pathogenic strains have suggested that genes involved in virulence are disproportionately associated with PAIs, a subclass of GIs [10], [15]. However, to date this association has never been quantified in a large-scale analysis encompassing multiple diverse pathogen genomes. In order to validate this observation, we used a dataset of 1568 virulence factors from the curated VFDB [19], [20] and quantified the occurrence of virulence factors in GIs for an initial group of 37 pathogens (representing 32 species and 23 genera that contained virulence factors from the VFDB and also had complete genome sequences available). To prevent circular logic where known PAIs are defined by the presence of virulence factors, and virulence factors are, therefore, found predominately in PAIs, we defined GIs based on attributes that are independent of such gene content or prior knowledge in the literature. We used three GI prediction methods that were used previously for other analyses of GIs and are considered effective methods for identifying GIs on a high-throughput scale [17], [18].

For our first analysis, a GI was defined as a region consisting of eight or more open reading frames (ORFs) with dinucleotide bias (calculated as the frequency of dinucleotides in a cluster of ORFs compared to the entire genome) as predicted by IslandPath-DINUC [17], [28]. This GI prediction method is noted for having more sensitivity/recall in predicting GIs versus other methods studied in an evaluation of GI predictors [18]. Consistent with previous anecdotal reports, our analysis indicated that GIs indeed contain a significantly higher proportion of virulence factors compared to non-GIs. On average for all the pathogens studied, 5.1% of genes in predicted IslandPath-DINUC islands are virulence factors, compared with 1.3% of genes outside of islands (*p* = 1.2E-135; Table 1). The significance of this is notable, given that such GI prediction methods tend to under-predict GIs [18]. Virulence factors were also enriched in GIs predicted using the more stringent and more specific/precise IslandPath-DIMOB method [17], [28], [29], which requires both dinucleotide bias and the presence of one or more mobility genes in the GI region (*p* = 1.3E-44; Table 1), as well as in GIs predicted using SIGI-HMM, which is based on an analysis of codon usage that removes ribosomal regions that may be falsely predicted as GIs (*p* = 4.9E-95) [30]. IslandPath-DIMOB and SIGI-HMM both have the highest overall accuracies for sequence composition-based prediction of GIs to date, but with lower sensitivity/recall than IslandPath-DINUC [18]. Regardless of which criterion was used, there was clearly a bias in terms of proportionately more virulence factors being located in predicted GI regions.

**Table 1:** Genomic Islands (GIs) contain higher proportions of virulence factors (VFs) than non–GI.

GI Identification Method	VF Dataset^a^	No. of VFs/Total no. of genes in GIs^b^ (%)	No. of VFs/Total no. of genes in non-GIs^b^ (%)	*p-*value^c^
**IslandPath-DINUC** d **(more sensitive method)**	All VFs	581/11437 (5.1)	1054/83161 (1.3)	1.2E-135
	Pathogen-associated^g^ VFs	160/10157 (1.6)	151/72201 (0.2)	2.8E-63
	“Common”^h^ VFs	421/11318 (3.7)	854/81791 (1.0)	6.4E-86
**IslandPath-DIMOB** e **(more specific method)**	All VFs	217/4601 (4.7)	1246/84832 (1.5)	1.3E-44
	Pathogen-associated^g^ VFs	58/4030 (1.4)	217/74311 (0.3)	3.7E-20
	“Common”^h^ VFs	159/4559 (3.5)	979/83391 (1.2)	1.2E-29
**SIGI-HMM** f **(more specific method)**	All VFs	387/7618 (5.1)	1039/80770 (1.3)	4.9E-95
	Pathogen-associated^g^ VFs	116/7029 (1.6)	149/71224 (0.2)	7.0E-51
	“Common”^h^ VFs	271/7616 (3.6)	890/79283 (1.1)	5.0E-51

**a** VFs are defined as those genes curated as being VFs according to the VFDB. Only VFs in the VFDB where GI predictions were available from IslandPath/SIGI-HMM were included in the analysis.

**b** Total number of genes in GIs varies according to the number of genomes used that contain pathogen-associated, “Common”, or both types of VFs.

**c** Fisher's exact test.

**d** GIs are defined as 8 or more consecutive ORFs with dinucleotide bias as predicted with IslandPath-DINUC.

**e** GIs are defined as 8 or more consecutive ORFs with dinucleotide bias plus presence of 1 or more mobility genes within the region as predicted with IslandPath-DIMOB.

**f** GIs are defined based on codon usage (removing regions like ribosomal operons) as predicted with SIGI-HMM. See text regarding the complementarity of the IslandPath-DIMOB and SIGI-HMM methods.

**g** Pathogen-associated VFs have homologs only in other pathogen genomes, at the similarity cut-off used (see Materials and Methods).

**h** “Common” VFs have homologs in both pathogens and non-pathogens (e.g. certain iron uptake systems, etc.) at the similarity cutoff used (see Materials and Methods).

A comparison of the virulence factors predicted to be in GIs by the different methods showed that while IslandPath-DINUC and SIGI-HMM agreed to a large extent (45% of virulence factors in predicted IslandPath-DINUC GIs were also in predicted SIGI-HMM GIs, and 67% of virulence factors in predicted SIGI-HMM GIs were also in predicted IslandPath-DINUC GIs), they are complementary approaches that each produce unique predictions (Figure S1). In fact, an analysis of the accuracy of combining the two methods together, using the approach published recently [18], revealed that the sensitivity/recall of these GI prediction methods combined increases notably (from 33% or 36% for SIGI-HMM or IslandPath-DIMOB, respectively, to 48% for the combined methods, with precision being maintained at 86%). However, for our analyses we wished to show that, regardless of the GI prediction method used, the results were significant; hence why we examined results using these three different GI prediction methods.

**Figure 1:**
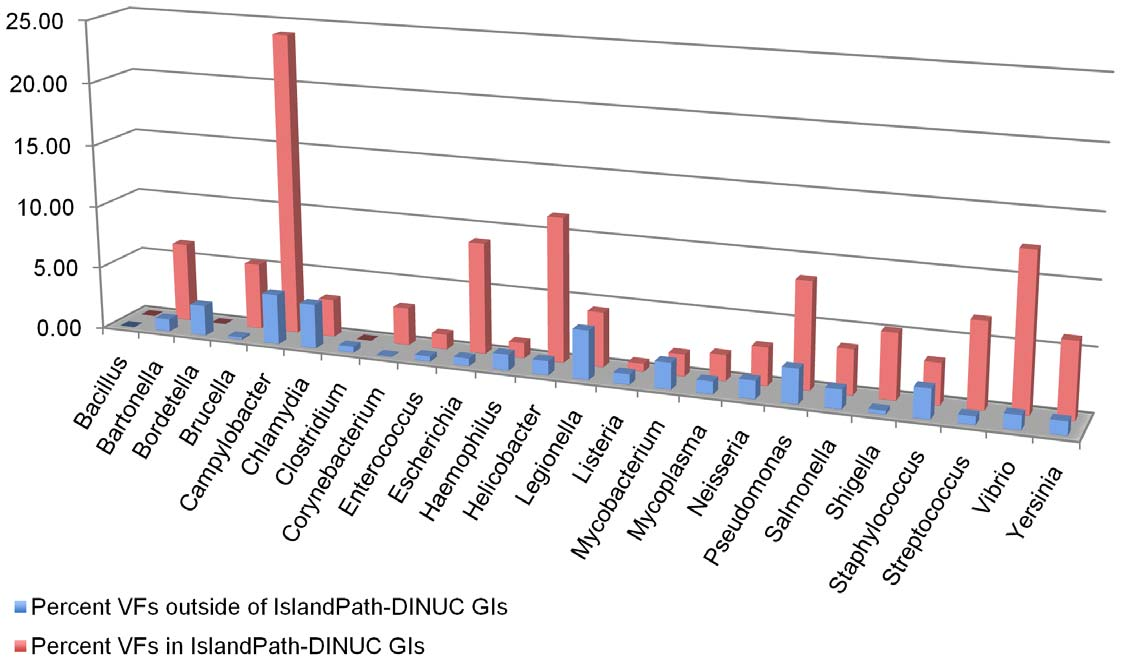
Enrichment of virulence factors (VFs) in GIs by pathogens. The proportion (%) of genes that are VFs in GIs (predicted by the IslandPath–DINUC method) for pathogens grouped by genus is shown in red, versus the proportion of genes that are VFs outside of GIs, which is shown in blue.

We then further examined the distribution of virulence factors in GIs by genus. We found that the enrichment of virulence factors in GIs is largely consistent with different pathogen lifestyles (Figure 1; see Figure S2 showing results for all three GI prediction methods). Pathogens capable of inhabiting multiple environmental niches, such as *Campylobacter*, *Vibrio*, *Escherichia* and *Pseudomonas spp*. exhibited the highest proportion of virulence factor genes in IslandPath-DINUC GIs. In comparison, for intracellular pathogens with limited horizontal gene transfer, such as *Chlamydia*, *Mycobacterium* and *Legionella*, we found no difference in the proportions of virulence factors inside and outside of GIs. Although none of the known *Bordetella* virulence factors resided in the predicted IslandPath-DINUC GIs, SIGI-HMM did identify the region spanning the *Bordetella* virulence factors (toxins and type III secretion components) as a predicted GI, further highlighting the complementary nature of the two approaches.

### Virulence factors in GIs tend to play more “offensive” roles

To study whether specific types of virulence factors are more likely to be associated with such probable horizontally transferred regions, we divided the virulence factors into 42 virulence-related categories adapted from the VFDB classification scheme, and examined the functional categories of virulence factors in GIs versus outside of GIs (with statistical corrections for multiple testing). We found that virulence factors over-represented in GIs are classified as type III secretion system and type IV secretion system components (including their corresponding effector proteins), as well as toxins and adherence factors (Table 2); such proteins comprise some of the most offensive weapons available to pathogens. These results are consistent with previous observations that type III and type IV secretion systems are closely associated with PAIs [16]. Type III and type IV secretion system genes were not, however, more significantly associated with GIs predicted specifically by IslandPath-DIMOB. It should be noted that such secretion systems may not have the types of mobile genes near them that the stringent DIMOB-based method detects, and that both IslandPath-DIMOB and SIGI-HMM, though more specific, have sensitivity in the ∼35% range [18]. Proteases and proteins involved in adherence, iron uptake, intracellular survival, capsule formation and antiphagocytosis were also preferentially associated with GIs (Table 2). The significant enrichment of “Unclassified” genes in GIs reconfirmed previous observations of a proposed large, novel gene pool that is associated with GIs [17].

**Table 2:** Classification of virulence factors (VFs) in Genomic Islands (GIs) and non–GIs.

VFDB Classification a	VFs in GIs^b^ (#)	Proportion of genes in GIs^c^ (%)	VFs in non-GIs (#)	Proportion of genes in non-GIs^d^ (%)	*p*-value^e^
Unclassifiedg,h (NA)	162	1.49	116	0.14	1.31E-75*
Type IV secretion system^f^ (O)	51	0.47	24	0.03	1.03E-28*
Type III secretion systeme,h(O)	97	0.89	154	0.19	1.12E-26*
Adherenceg,h (O)	107	0.98	195	0.24	8.17E-26*
Iron uptake (NS)	31	0.28	60	0.07	1.81E-07*
Intracellular survivalg,h (NS)	8	0.07	4	0.00	8.15E-05*
Toxin^g.h^ (O)	25	0.23	63	0.08	1.14E-04*
Capsule^g^ (D)	4	0.04	0	0.00	1.00E-03*
Protease^g^ (D)	5	0.05	4	0.00	8.77E-03*
Antiphagocytosis (D)	18	0.17	67	0.08	3.89E-02*
Immune evasion^h^ (D)	3	0.03	8	0.01	4.99E-01
Actin-based motility (O)	1	0.01	1	0.00	7.75E-01
Secretion system (other) (NS)	16	0.15	98	0.12	8.22E-01
Invasion (O)	2	0.02	7	0.01	8.22E-01
IgA1 Protease (D)	1	0.01	2	0.00	8.22E-01
Magnesium uptake (NS)	1	0.01	2	0.00	8.22E-01
Motility (NS)	7	0.06	67	0.08	1.00E+00
Exoenzyme (NS)	2	0.02	31	0.04	1.00E+00
Endotoxin (NS)	3	0.03	29	0.04	1.00E+00
Regulation (R)	3	0.03	26	0.03	1.00E+00
Type II secretion system (NS)	0	0.00	22	0.03	1.00E+00
Stress protein (D)	1	0.01	11	0.01	1.00E+00
Cellular metabolism (D)	0	0.00	8	0.01	1.00E+00
Enzyme (NS)	0	0.00	8	0.01	1.00E+00
Cell wall (NS)	1	0.01	6	0.01	1.00E+00
Biofilm formation (D)	0	0.00	4	0.00	1.00E+00
Molecular mimicry (D)	0	0.00	4	0.00	1.00E+00
Intracellular growth (NS)	0	0.00	3	0.00	1.00E+00
Plasminogen activator (O)	0	0.00	3	0.00	1.00E+00
Serum resistance (D)	0	0.00	3	0.00	1.00E+00
Biosurfactant (NS)	0	0.00	2	0.00	1.00E+00
Pigment (O)	0	0.00	2	0.00	1.00E+00
Proinflammatory effect (NS)	0	0.00	2	0.00	1.00E+00
Anti-proteolysis (D)	0	0.00	1	0.00	1.00E+00
Bile resistance (D)	0	0.00	1	0.00	1.00E+00
Complement Protease (D)	0	0.00	1	0.00	1.00E+00
Complement resistance (D)	0	0.00	1	0.00	1.00E+00
Heat-shock protein (NS)	0	0.00	1	0.00	1.00E+00
Manganese uptake (NS)	0	0.00	1	0.00	1.00E+00
Nutrient acquisition (NS)	0	0.00	1	0.00	1.00E+00
Peptidase (D)	0	0.00	1	0.00	1.00E+00
Resistance to antimicrobial peptides (D)	0	0.00	1	0.00	1.00E+00
**TOTALS**	**549**		**1045**		

**a** VFs are defined as those genes curated as being VFs according to the VFDB. Only those VFs in the VFDB where GI predictions were available from IslandPath were included in the analysis. VFs are also categorized, according to the VFDB, as O = Offensive; D = Defensive; NS = Nonspecific; R = Regulation; NA = Not Available.

**b** Number of VFs in GIs predicted with IslandPath-DINUC (more sensitive method).

**c** Proportion of genes in GIs that are VFs.

**d** Proportion of genes in non-GIs that are VFs.

**e** Fisher's exact test with Benjamini-Hochberg correction for multiple testing. Asterisks indicate statistical significance (*p*-value<0.05).

**e** Includes Type III secretion system genes and Type III translocated proteins.

**f** Includes Type IV secretion system genes and Type IV secretory proteins.

**g** Categories of VFs that were also statistically significant with the IslandPath-DIMOB dataset.

**h** Categories of VFs that were also statistically significant with the IslandPath-SIGI-HMM dataset.

We used the VFDB division of virulence factors among four categories – “offensive”, “defensive”, “nonspecific”, and “regulatory” – to quantitatively test our hypothesis that virulence factors in GIs play more offensive roles. It is notable that regardless of the GI detection method used, virulence factors classified as offensive by the VFDB (i.e. involved in active invasion of the host or that directly cause damage to the host) were very significantly associated with GIs (*p* = 3.5E-37; using IslandPath-DINUC). In contrast, defensive virulence factors (i.e. involved in passive defense/evasion of the host) were associated with GIs to a much lesser degree using the IslandPath-DINUC data set (*p* = 0.04), and the association was not statistically significant using the DIMOB and SIGI-HMM data sets (Table 3). Nonspecific virulence factors (i.e. neither offensive nor defensive, or both depending on context) were also significantly associated with GIs. Notably, no functional classes of virulence factors, according to the VFDB classification system, were more prevalent outside of GIs at a statistically significant level.

**Table 3:** Virulence factors (VFs) in genomic islands (GIs) play more “offensive” roles.

VF Type	Proportion of genes in DINUC GIs (%)	Proportion of genes in non-DINUC regions (%)	*p*-value^a^	Proportion of genes in DIMOB GIs (%)	Proportion of genes in non- DIMOB regions (%)	*p*-value^a^	Proportion of genes in SIGI-HMM GIs (%)	Proportion of genes in non- SIGI-HMM regions (%)	*p*-value^a^
Offensive	2.53	0.97	3.50E-37	1.64	1.15	4.98E-03	5.51	1.01	6.49E-144
Defensive	0.26	0.16	3.69E-02	0.18	0.17	8.52E-01	0.22	0.17	3.17E-01
Nonspecific	0.96	0.34	1.60E-16	0.57	0.41	1.16E-01	1.26	0.40	2.81E-19
Regulation	0.03	0.04	7.93E-01	0.00	0.04	1.70E+01	0.05	0.04	5.45E-01

**a** Fisher's exact test.

### Both pathogen-associated virulence factors and “common” virulence factors (having homologs in both pathogens and non-pathogens) are associated with GIs

We hypothesized that virulence factors found predominately in pathogens are more directly involved in pathogenicity (i.e. directly cause damage to the host and/or are sufficient to cause disease), and that, in contrast, virulence factors with identifiable homologs in both pathogens and non-pathogens are more likely to facilitate host interactions. To examine this further, we performed a sequence similarity search against 631 completely sequenced bacterial genomes for 298 pathogens and 333 non-pathogens to classify each virulence factor in VFDB as “pathogen-associated” – having homologs only in pathogens – or “common” – having homologs in both pathogens and non-pathogens (see Materials and Methods). Of 2285 virulence factors, 515 (23%) were pathogen-associated and 1770 (77%) were “common”.

We investigated the relationship between GIs and pathogen-associated virulence factors by quantifying the proportion of pathogen-associated virulence factors and “common” virulence factors in GIs relative to all genes in GIs. This analysis was performed using only those virulence factors from organisms with completely sequenced genomes. Regardless of the GI prediction criteria used (IslandPath-DINUC, IslandPath-DIMOB or SIGI-HMM), both pathogen-associated and “common” virulence factors were present in higher proportions in GIs than outside of GIs for the pathogens examined (Table 1). While pathogen-associated virulence factors might be expected to be associated with GIs, since many are new genes that have been recently acquired, it is notable that “common” virulence factors with potentially older evolutionary origins have also been retained in GIs across multiple genera.

Our classification of virulence factors as pathogen-associated or “common” allowed for some notable observations regarding common mechanisms of virulence. Analysis of VFDB functional classes indicated that pathogen-associated virulence factors are disproportionately toxins or involved in type III and type IV secretion systems. It is noteworthy that some classes of toxins were restricted to pathogens yet were present in multiple diverse genera. Some examples include toxins with adenylate cyclase activity (anthrax toxin edema factor from *Bacillus anthracis* and exoenzyme Y from *Pseudomonas aeruginosa,* which can be found in four genera) and toxins with ADP-ribosyltransferase activity (pertussis toxin, cholera toxin, and *P. aeruginosa* exoenzyme S and exoenzyme T, all present in four or more genera). In contrast, “common” virulence factors were involved in motility, antiphagocytosis, iron uptake, endotoxin, and type II secretion system functions (see Table 4 for a listing of the subset of categories that were statistically significant; Table S1 for a complete list of categories). Overall, pathogen-associated virulence factors were significantly disproportionately classified as offensive by VFDB (*p* = 1.77E-22), while “common” virulence factors tended to have defensive or nonspecific functions (*p* = 2.07E-08 and *p* = 7.88E-07, respectively).

**Table 4:** Statistically significant categories of virulence factors (VFs) that are Pathogen-associated or “Common” to both pathogens and non-pathogens.

VFDB Classification^a^	Pathogen-associated^b^ (%)	“Common”^c^ (%)	*p*-value^d^
Categories with a higher percentage of Pathogen-associated VFs			
Toxin (O)	79 (15.28)	58 (3.27)	1.84E-18*
Type III secretion system (O)	117 (22.63)	175 (9.87)	1.02E-11*
Type IV secretion system (O)	32 (6.19)	51 (2.88)	4.77E-03*
Categories with a higher percentage of “Common” VFs			
Motility (NA)	0 (0)	75 (4.23)	9.95E-08*
Antiphagocytosis (D)	6 (1.16)	105 (5.92)	1.13E-05*
Iron uptake (NS)	5 (0.97)	92 (5.19)	2.51E-05*
Endotoxin (NS)	0 (0)	32 (1.80)	2.98E-03*
Type II secretion system (NS)	0 (0)	22 (1.24)	4.24E-02*

**a** VFs are defined as those genes curated as being VFs according to the VFDB. VFs are also categorized, according to the VFDB, as O = Offensive; D = Defensive; NS = Nonspecific; R = Regulation; NA = Not Available.

**b** Pathogen-associated VFs have homologs only in other pathogen genomes, at the similarity cut-off used (see Materials and Methods).

**c** “Common” VFs have homologs in both pathogens and non-pathogens (e.g. certain iron uptake systems, etc.) at the similarity cutoff used (see Materials and Methods).

**d** Fisher's exact test. Only those categories with statistical significance (*p*-value<0.05) are listed.

In an extended analysis examining all genes in the available 631 bacterial genomes, we determined that 14% of genes in pathogen genomes were pathogen-associated, and 19% of genes in non-pathogen genomes had homologs exclusively in non-pathogens using our criteria (see Materials and Methods). Both pathogen-associated and non-pathogen-associated genes occurred significantly more frequently in GIs than non-GIs (*p≈*0 for both, regardless of which prediction criteria were used). This supports our previously published observation that species or family-specific genes tend to be more commonly found in GI regions because of a probable larger gene pool associated with such mobile elements [17]. It should not distract, however, from our earlier observation that virulence factors in general were clearly disproportionately associated with GIs, including those with homologs in non-pathogens.

Collectively, these results confirm previous anecdotal reports that virulence factors are more common in GIs, supporting the important role of GIs in pathogen evolution. The results also further suggest that offensive and pathogen-associated (i.e. potentially more virulence-specific) virulence factors are more likely to have been recently acquired (in the time scale detected by GI analysis), versus those involved in more defensive or passive host-association functions.

### CRISPRs are associated with GIs

Clustered regularly interspaced short palindromic repeats (CRISPRs) are genetic elements that have been identified in approximately 40% and 90% of Bacteria and Archaea genomes, respectively [31]. A CRISPR consists of several identical repeats, separated by non-identical spacer sequences [32]. These repeat and spacer sequences typically range in size from 25–40 base pairs long, while the number of repeats in a single CRISPR varies widely from 2 to 250 [31]. Recent research has shown that these elements, along with CRISPR associated (CAS) genes, are involved in a silencing mechanism that can provide protection against phage and possibly other mobile elements [33], [34]. Furthermore, the phylogenetic profiles of CAS genes suggest that CRISPR systems could be primarily transferred by horizontal gene transfer [35]–[37].

Although CRISPRs have been identified on ten megaplasmids [35] and within two prophages in *Clostridium difficile* [38], a large scale analysis of CRISPRs and GIs has not been conducted. To evaluate if an association exists, we obtained 1043 predicted CRISPRs for 355 species from the CRISPRdb (http://crispr.u-psud.fr/crispr/) [31]. In our analysis, CRISPRs were found to be over-represented within GIs predicted by all three methods, with approximately twice as many CRISPRs located in GIs than expected (Table 5). Furthermore, the over-representation of CRISPRs within GIs was statistically significant using chi-squared analysis.

**Table 5:** Over-representation of CRISPRs in GIs.

	IslandPath-DINUC	IslandPath-DIMOB	SIGI-HMM
Number of bacterial genomes^a^	245	237	213
Number of GIs	23889	6158	7529
Proportion of genome in GIs (%)	11.0	4.2	3.1
Total number of CRISPRs	684	661	607
Expected number of CRISPRs in GIs	75	28	19
Observed number of CRISPRs in GIs	145	66	43
***p*-value^b^**	1.4E-17	6.5E-14	1.4E-08

**a** Number of bacterial genomes for which both CRISPRs and GIs could be predicted.

**b** Chi-squared test includes number of observed and expected CRISPRs outside of islands (data not shown).

Given the relationship between phage and CRISPRs, we investigated the contribution of phage genes to GIs in a separate study examining all GIs predicted by IslandPick (a comparative genomics-based GI prediction method [18], [29]), SIGI-HMM or IslandPath-DIMOB (the two most accurate sequence composition-based methods which could be more widely applied to genomes since they do not require comparative genomes). The frequency of genes in GIs with the word “phage” occurring in the annotation was enumerated (henceforth referred to as “phage genes”) and compared to the number of phage genes outside of GIs. GIs in prokaryotic genomes were significantly enriched for genes with a phage annotation (6990 observed; 1264 expected; p≈0), supporting the idea that a large number of GIs are prophage regions. GIs that contained CRISPRs also showed over-representation of phage genes (p = 5.7E-05).

### Sampling bias in the currently available dataset of bacterial genomes is not a major contributing factor to our observations

One potential source of bias with the functional category analysis is that the taxonomical distribution of the genomes sequenced to date is uneven. In particular, some pathogens are over-represented by multiple strains while certain, predominately non-pathogenic, taxa are sparsely represented. To reduce redundancy and bias in the whole genome dataset, we selected a subset of pathogen and non-pathogen genomes with a minimum evolutionary distance (substitutions/site) of 0.05 (adapted from a recent phylogenetic analysis [39]). This essentially reduced the number of pathogen genomes that were highly similar (e.g. multiple strains of a pathogen) and thus reduced sampling bias. When this less-biased genome dataset was analyzed again using the same classification schemes and methods as described above, no significant differences in results were observed (data not shown), with still highly significant *p-*values for our statistics, indicating that the sampling bias in sequenced genomes is not a major contributing factor to our observations.

## Discussion

Collectively, our data provide large scale, quantitative measurements regarding trends in virulence across multiple genera that are either newly identified or have been previously stated for selected pathogens. In our study, we confirm previous studies of closely related species reporting that virulence factors are commonly found in GIs, and we find that these virulence factors are disproportionately involved in more offensive versus defensive functions. The association of virulence factors with GIs holds true regardless of whether we use the more sensitive IslandPath-DINUC method for GI prediction, or more specific methods, such as IslandPath-DIMOB and SIGI-HMM. It should be emphasized that the methods used will not detect some GIs – such as those that have been acquired from genomes with similar sequence composition, or more ancient GI acquisition events that may have ameliorated to the host genome sequence composition over time. Therefore, these GI prediction methods will tend to under-predict GIs. However, even with this under-prediction, we notably never observe a statistically significant association of virulence factors with regions outside of GIs for any virulence factor class. These results also hold true for GIs defined using whole-genome comparative methods. We analyzed an alternate set of GIs defined by Vernikos and Parkhill (2008) as genomic regions with limited phylogenetic distribution consistent with recent acquisition [40], and found that there are indeed more virulence factors in this set of GIs compared to outside of such GIs (p = 9.5E-160) (See Text S1 and Table S2). These data strongly support the important role of GIs in pathogen evolution.

We used the set of virulence factors in the VFDB. This is a well-established, published set based on experimentally demonstrated virulence factors extracted from the literature and supplemented with comparative genomics. Prior to performing this study, we evaluated several virulence factor repositories, including the PRINTS database (http://www.jenner.ac.uk/BacBix3/PPprints.htm), the Toxin and Virulence Factor Database (TVFac) at Los Alamos National Laboratory, MvirDB (http://mvirdb.llnl.gov) [41], and looked also at COG [42] classifications for virulence factors. VFDB was found to be the most comprehensive and had the highest quality with its curated dataset and virulence-guided classification system. To further verify our results using an independently derived set of virulence factors, we examined virulence proteins from Swiss-Prot [43] and found their association with genomic islands to also be significant (p<8.4E-04 for all GI prediction methods) (See Text S1 and Table S3). Using the VFDB classification scheme, we note that pathogen-associated virulence factors have more offensive functional roles compared to “common” virulence factors, which are likely to be so-called “host interaction factors” with nonspecific or defensive functions. In particular, type III and type IV secretion components and toxins tend to be over-represented in GIs. The type III secretion system is a well-studied virulence mechanism; type IV secretion systems are implicated in conjugation of DNA as well as the delivery of effector molecules to host cells [44], once again highlighting the contextual nature of virulence. These observations suggest that pathogenicity, as opposed to host interaction, is more often a recently developed phenomenon in a species (on an evolutionary time scale detected by GI analysis).

GIs appear to provide a critical flexible mechanism that allows a bacterium to adapt and develop increased, invasive infection in the host. Several evolutionary models have been proposed to explain how virulence factors are maintained and these models are consistent with the importance of GIs (and related phage) in pathogen evolution. Smith [45] proposed that in a pathogen population, there are a small number of “cheaters” that themselves do not possess certain extracellularly-acting virulence factors but benefit from the effect of these virulence factors released by the non-cheater strains. Without the virulence factors, the cheater strains are metabolically more fit than the non-cheaters, and therefore their number would increase in the population over time. However cheaters, due to their lack of virulence factors, have decreased infectiousness and Smith proposed that horizontal gene transfer was a possible mechanism to minimize “cheater” strains and restore infectiousness in the pathogen population. As a result, certain virulence factors are maintained on mobile elements, including GIs, which are thought to be related to phage [17]. It is worth noting that PSORTb analysis of subcellular localization [46] indicates that predicted extracellular proteins are over-represented in pathogen-associated genes for Gram-positives (there is better prediction of extracellular factors for Gram-positives versus Gram-negatives) (data not shown). In a second proposed model, Sokurenko and colleagues adopted the classical source-sink model of population genetics to describe virulence evolution [47]. For opportunistic pathogens, the environmental reservoir represents a self-sustainable source whereas the opportunistic infection represents a venture into a sink. They proposed that acquisition of PAIs as a mechanism to facilitate adaptation of the source to sink transition whereas the loss of PAIs accompanies the sink to source transition. However, since possessing a PAI can significantly increase the pathogen's fitness in the sink, which in turn increases the back flow of PAI-possessing strains into the source population, virulence factors in PAIs can be maintained despite the negative fitness value of virulence factors in the environment. It is notable that, in our study, many of the over-represented virulence factors in GIs are involved in active invasion that harm the host in some way and there is no obvious functionality for these virulence factors outside of the host environment. Therefore, maintaining these virulence factors on GIs, and likely other mobile elements like phages and plasmids, appears to provide important evolutionary flexibility for these pathogens.

Our investigation of putative pathogen-associated virulence factors reveals several universal strategies adopted by pathogens to gain access to and colonize privileged sites in hosts, i.e. the use of toxins and host contact-dependent secretion mechanisms. These strategies appear to be relatively absent in non-pathogenic strains which also typically do not elicit a strong inflammatory response [48]. The recent publication of the *Hamiltonella defensa* genome highlights the complexity inherent in virulence [49]. *H. defensa* is an endosymbiont that protects its aphid host from attack by parasitoid wasps. Numerous homologs of known virulence factors, including genes for toxins, effector proteins and two type III secretion system components are present in its genome, resulting in speculation that the encoded virulence factors play a role in symbiosis. However, it should be noted that the type III secretion system did not appear to be active based on proteomics analysis of intact *H. defensa* cells from whole insects – only one of the type III secretion proteins were recovered and none of the effectors were expressed [49]. We therefore argue that *H. defensa*'s type III secretion system may be required for its role as a wasp pathogen rather than having new roles related to symbiosis.

Several inactivated toxins (toxoids) such as pertussis toxin and diphtheria toxin have been used successfully as vaccines against the associated pathogen [50]. Epidemiological evidence has indicated that these vaccines, which induce antitoxin immunity, resulted in a significant reduction in the virulent form of these pathogens [51], [52]. These results, combined with the observation that many of these virulence factors are not part of the core pathogen genomes, suggest that if we put selection pressure on virulent specific antigens, we may be able to effectively reduce the number of pathogens carrying these genes, and hence provide selection for pathogens to evolve into less virulent forms. Our study supports observations that several virulence factors used in successful vaccinations are indeed specific to, or associated with, pathogens. Additional review of the pathogen-associated virulence factors we have identified in this analysis shows that some are protective either on their own or in combination with other pathogen-associated virulence factors in an animal model of infection. However, not all have been tested and clearly it would be prudent to examine the efficacy of other strongly pathogen-associated genes that have not yet been investigated for their effectiveness in vaccines. Antigens that are common to both pathogens and commensals may, on average, be less likely to elicit strong immunogenic responses. Our study provides lists of pathogen-associated genes that may encode good candidates for vaccine development or anti-virulence drug development [53], [54].

In addition, we provide supporting evidence that CRISPRs are over-represented within GIs and therefore, are likely being horizontally transferred. Several studies have shown that CRISPRs can derive from both viruses and plasmids [34], [55]. We provide evidence indicating that some GIs containing CRISPRs are likely to be prophages, lending further support to observations that phages can carry CRISPR sequences within their genomes. CRISPRs have been proposed to be beneficial to bacteria, facilitating defense against viral infections. However, this work indicates that phage more directly may be hosting these repeat elements, to help avoid additional phage entering a genome that already hosts a given prophage. Most of this data is speculative, but it does suggest that understanding the association of CRISPRs with islands and phages is important, given the association of GIs with virulence and other microbial adaptations of medical and industrial importance.

It should be noted that our study has several limitations. We are significantly limited by the number and diversity of genome sequences and known virulence factors currently available. However, we felt that the diversity of species whose genome sequences were available was now sufficient to provide an early sense of the degree to which certain genes were pathogen-associated since multiple well studied pathogens, with closely related non-pathogenic relatives, had complete genomes available from diverse phyla. We also repeated our analyses involving hundreds of genomes, taking into account the phylogenetic distance between species to reduce the redundancy of the genomes dataset in order to reduce potential sampling biases. We obtained similar results, with the same statistically significant observations, with this pared down dataset. Regardless, clearly this analysis, or a similar type of analysis, bears repeating as the number of genome sequences available increases. Future analyses will need to increasingly account for non-pathogens that may have recently evolved from pathogens and that may still contain remnants of virulence factors. Also, there is some uncertainty regarding whether a given organism (such as a novel, poorly understood marine microbe) is a pathogen or not, since any host interactions may be unknown. The contextual nature of pathogenicity (for example, how an organism can be a pathogen in one species and not in another) complicates analysis and will need to be further considered. Of course, exceptions to these trends will also always be found. However, by examining many diverse species as a group, we do somewhat overcome uncertainty or pathogen classification errors in a few cases by the sheer numbers of organisms we analyzed and the diversity of phyla examined. Our investigations were also limited by the cutoffs used in the analysis of similarity between sequences. We chose cutoffs for similarity that did not produce a notably different result from cutoffs slightly above or below it (data not shown). However, any hard cutoff is not perfect and so we encourage, and are performing ourselves, further manual inspection of results for a given gene identified as pathogen-associated before pursuing further in depth analysis of the gene of interest. It should also be taken into consideration that some proteins, such as type III secretion system effectors, may appear to be more pathogen-associated simply because there are less constraints on their sequence and they have diverged in sequence more rapidly. Finally, we also investigated the utility of different gene function classification systems in this analysis, like COG, SUPERFAMILY, PRINTS, and the VFDB. It became clear over the course of this study that general classification systems such as COG do not perform well in detecting trends in virulence since the classification system does not include most virulence factors at all and does not have virulence-associated categories. The VFDB, with its curated dataset and virulence-guided classification system, was found to be the most effective. However, there are still some VFDB classifications that could benefit from more curation – for example the Type III secretion system component classification could be improved further. Even though virulence is a complex phenomenon, more effort should be made to build upon such efforts and develop a high quality ontology that is relevant to virulence, to complement other ontology efforts.

Even with all of the limitations in our analysis described above, the criteria we used clearly identify genes and gene categories that have a notable pathogen association. Genes that are present in multiple pathogens of different genera, but not present in non-pathogens of these same genera, are certainly worthy of being described as being pathogen-associated. Such genes warrant further study as part of the efforts to develop more anti-infective therapies and vaccines, as well as for their role in virulence in general. Of particular interest are the many conserved hypothetical genes shared across multiple diverse genera that were identified as pathogen-associated. Our analyses of GIs likewise provide strong evidence that these genomic regions can, on average, play a critical role in virulence. Further examination of the origins of such pathogen-specific genes and GIs, their relationship to phage, and how their products integrate with the existing cellular network, could provide enlightening insight into global trends in the evolution of virulence.

In conclusion, our analyses of a curated dataset of virulence factors and pathogen-associated genes suggest that such genes are, on average, more associated with GIs versus non-GI regions. Our collective results also further suggest that offensive and virulence-specific virulence factors in bacterial pathogens are more likely to be associated with GIs, versus virulence factors with homologs in non-pathogens that tend to be involved in more passive host-association functions. Though there are certain bacteria (such as obligate intracellular pathogens) that are exceptions, this work clearly demonstrates the strong role of GIs in the evolution of virulence and provides the first systematic analysis of this trend across diverse genera. We provide evidence that certain types of virulence factors, such as components of host contact-dependent secretion systems and certain classes of toxins, are quite selectively pathogen-associated. Additionally, we provide whole genome datasets of pathogen-associated genes in a set of completely sequenced bacterial genomes. Such pathogen-associated genes, in particular those found in diverse pathogens but not in non-pathogens in the same genera, warrant further study for their potential role in virulence, as well as for their potential as anti-infective drug targets or vaccine components.

## Materials and Methods

### Virulence factors and pathogen-associated genes

A dataset of 2293 virulence factors (from 37 pathogenic bacterial species) was obtained from the VFDB (http://www.mgc.ac.cn/VFs/) [19], [20] in March 2008. Each virulence factor from the VFDB dataset was identified as pathogen-associated (found predominately in pathogens), or “common” (found in both pathogens and non-pathogens) through a BLAST similarity search against the deduced proteomes of 298 pathogenic and 333 non-pathogenic sequenced prokaryotic genomes obtained from the National Center for Biotechnology Information (NCBI) FTP site in March 2008. Both chromosome and plasmid replicon types were included in the analysis. An E-value cutoff of 10^-7^ was selected to exclude distant homologs. In an initial investigation we examined more and less stringent cutoffs of 10^−12^ and 10^−5^, respectively, and found that the vast majority of trends analyzed still hold when these other cutoffs were used. Pathogen, non-pathogen, and host-associated status for each genome were initially obtained from the summary page of “Complete Microbial Genomes” at NCBI (http://www.ncbi.nlm.nih.gov/genomes/lproks.cgi) [56] and then manual curation for data quality and overall completeness was performed on this dataset. We also identified pathogen-associated, “common”, and non-pathogen-associated (genes found predominately in non-pathogens) genes for each gene in the sequenced genomes in a similar manner as described above. These data sets are available for download (http://www.pathogenomics.sfu.ca/pathogen-associated/). Additionally, to reduce redundancy and bias in this whole genome dataset (multiple genome sequences from a particular genera or species), we repeated the analysis using a subset of genomes with a minimum evolutionary distance (substitutions/site) of 0.05 (based on a recent phylogenetic analysis [39]).

### Virulence factors and pathogen-associated genes in GIs

To quantify the number of virulence factors in GIs, we used a subset of virulence factors from the VFDB (described above) for which fully-sequenced genomes are available. A total of 1565 virulence factors were used from 28 different genomes. We quantified the occurrence of virulence factors in GIs, where GIs were defined as either 1) IslandPath-DINUC: a region consisting of 8 or more ORFs with dinucleotide bias (a more sensitive method for GI detection), or 2) IslandPath-DIMOB: a region of 8 or more ORFs with dinucleotide bias plus the presence of one or more mobility genes (a more specific method of GI detection [17]), or 3) SIGI-HMM: DNA regions showing atypical codon usage based on HMM analysis [30]. The IslandPath software application [28] was subjected to slight modifications to improve predictive accuracy [18], and GI predictions are available for IslandPath-DIMOB and SIGI-HMM methods through IslandViewer [29]. Predictions were made for chromosomal and plasmid DNA. Note that there are many more genes in general outside of GIs than in GIs for any genome, so we normalized the proportions of virulence factor genes inside or outside islands as a function of the total number of genes inside and outside of such GI regions. The occurrence of pathogen-associated genes in GIs for sequenced prokaryotic genomes were counted in a similar manner as described in the above section, again examining the proportions of such genes as a function of the total number of genes in GI or non-GI regions.

### Characterization of features and functional classes of virulence factors and pathogen-associated genes

The VFDB uses keywords describing virulence-related functions to assign virulence factors to one of four broad classes: “offensive”, “defensive”, “regulation” and “nonspecific”. We adapted and curated the VFDB classification, assigning unclassified keywords (and their related virulence factors) to one of the four classes, and reclassifying virulence factors in cases where we disagreed with the current annotation. For example, in the VFDB, lipopolysaccharide (LPS) from *Pseudomonas aeruginosa* is mapped to the terms “endotoxin” and “adherence”. Since “adherence” is considered an “offensive” keyword, LPS is listed as an “offensive” virulence factor. We reclassified LPS to be nonspecific in keeping with its general function. The revised classification is available in Table 2.

### Statistical analyses

Statistics for over-representation of virulence factors in GIs were performed by tabulating the number of virulence factors in GIs, total number of genes in GIs, number of virulence factors outside of GIs, and total number of genes outside of GIs in a 2×2 contingency table, and then using the Fisher's Exact Test. Similar statistical analysis was done for functional classification of genes in islands, where the number of genes in each VFDB category was used in the calculation. Over- or under-representation of VFDB functional classifications of pathogen-associated and “common” virulence factors was done by comparing the number of pathogen-associated genes in a given category against “common” genes in the same category. Since multiple categories are examined simultaneously, the Benjamini and Hochberg False Discovery Rate correction for multiple testing was performed for all functional category analyses. We considered *p*-values smaller than 0.05 to be significant. All statistics were calculated using the *R* statistics package.

### Over-representation of CRISPRs within GIs

Predicted CRISPRs were obtained from the CRISPRdb (http://crispr.u-psud.fr/crispr/) [31]. This database contained 1043 CRISPRs for 355 species (306 Bacteria and 49 Archaea). The coordinates of bacterial CRISPRs were searched among GIs in species for which GIs could be predicted by IslandPath-DINUC, IslandPath-DIMOB, or SIGI-HMM. We tabulated the number of CRISPRs in GIs and compared it to the expected number based on genome size and CRISPR frequency. To approximate the contribution of phage to GIs, the frequency of genes in GIs with “phage” occurring in the annotation (referred to as “phage genes”) was calculated and compared to the frequency of phage genes outside of GIs. We further extended this analysis to determine the over-representation of phage genes in GIs containing CRISPRs compared to non-GI regions. The significance of over-representation was determined using a chi-squared test.

## Supporting Information

Figure S1 Venn diagram showing the overlap of virulence factors in GIs predicted using three methods: IslandPath-DINUC, IslandPath-DIMOB, and SIGI-HMM.
(0.22 MB TIF)

Figure S2 Proportion of genes (%) that are virulence factors (VFs) inside versus outside of (A) IslandPath-DINUC, (B) IslandPath-DIMOB, and (C) SIGI-HMM GIs. Pathogens having GI predictions are grouped by genus.
(1.22 MB TIF)

Table S1 Complete list of VFDB functional classifications of pathogen-associated and “common” virulence factors from the VFDB. Only statistically significant categories are shown in Table 3.
(0.07 MB DOC)

Table S2 Analysis of VFDB virulence factors in a set of GIs derived from whole-genome comparisons by Vernikos and Parkhill (2008).
(0.06 MB DOC)

Table S3 Enrichment of Swiss-Prot-derived virulence proteins in GIs.
(0.03 MB DOC)

Text S1 Supplemental methods.
(0.03 MB DOC)
