# Supplementary figures and images for: The Association of Virulence Factors with Genomic Islands
Source: PLoS One. 2009 Dec 1;4(12):e8094. doi: 10.1371/journal.pone.0008094 (PMC2779486; doi:10.1371/journal.pone.0008094)

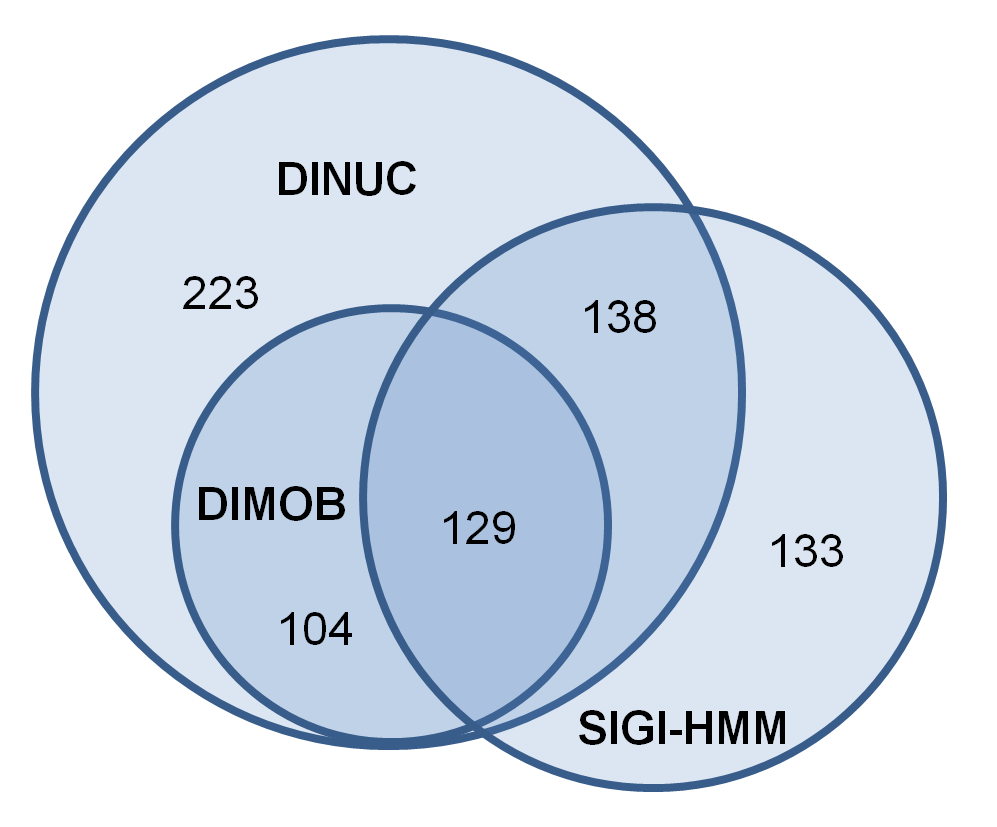

Supplement: Figure S1 — Venn diagram showing the overlap of virulence factors in GIs predicted using three methods: IslandPath-DINUC, IslandPath-DIMOB, and SIGI-HMM. (0.22 MB TIF) [file pone.0008094.s001.tif]

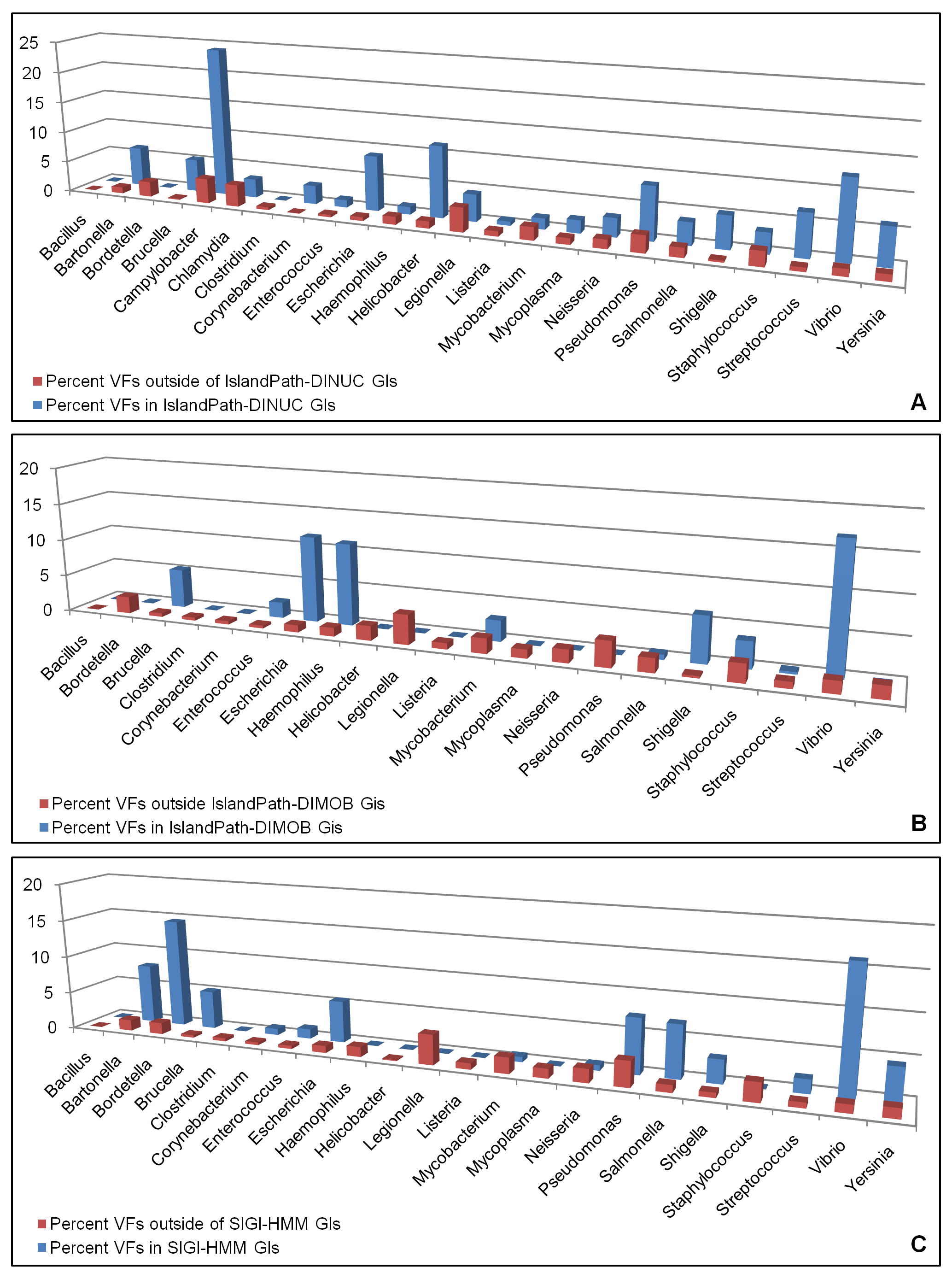

Supplement: Figure S2 — Proportion of genes (%) that are virulence factors (VFs) inside versus outside of (A) IslandPath-DINUC, (B) IslandPath-DIMOB, and (C) SIGI-HMM GIs. Pathogens having GI predictions are grouped by genus. (1.22 MB TIF) [file pone.0008094.s002.tif]
