# Supplementary material for: The Association of Virulence Factors with Genomic Islands
Source: PLoS One. 2009 Dec 1;4(12):e8094. doi: 10.1371/journal.pone.0008094 (PMC2779486; doi:10.1371/journal.pone.0008094)
Supplement: Table S1 — Complete list of VFDB functional classifications of pathogen-associated and “common” virulence factors from the VFDB. Only statistically significant categories are shown in Table 3. (0.07 MB DOC) [file pone.0008094.s003.doc]

## Table S1 - VFDB classification of Pathogen-associated and “Common” VFs

| **VFDB Classification^a^** | **Pathogen-associated VFs (%)^b^** | **“Common” VFs (%)^b^** | ***p*-value^c^** |
| --- | --- | --- | --- |
| Categories with a higher percentage of Pathogen-associated VFs |  |  |  |
| Toxin (O) | 79 (15.28) | 58 (3.27) | 1.84E-18* |
| Type III secretion system^d^ (O) | 117 (22.63) | 175 (9.87) | 1.02E-11* |
| Type IV secretion system^e^ (O) | 32 (6.19) | 51 (2.88) | 4.77E-03* |
| Plasminogen activator (O) | 2 (0.39) | 1 (0.06) | 4.06E-01 |
| Anti-proteolysis (D) | 1 (0.19) | 0 (0.00) | 4.97E-01 |
| Antivirulence (R) | 1 (0.19) | 0 (0.00) | 4.97E-01 |
| Actin-based motility (O) | 1 (0.19) | 1 (0.06) | 7.34E-01 |
| Proinflammatory effect (NS) | 1 (0.19) | 1 (0.06) | 7.34E-01 |
| Capsule (D) | 1 (0.19) | 3 (0.17) | 1.00E+00 |
| Unclassified (NA) | 151 (29.21) | 505 (28.48) | 1.00E+00 |
| Categories with a higher percentage of "Common" VFs |  |  |  |
| Motility (NS) | 0(0.00) | 75 (4.23) | 9.95E-08* |
| Antiphagocytosis (D) | 6 (1.16) | 105 (5.92) | 1.13E-05* |
| Iron uptake (NS) | 5 (0.97) | 92 (5.19) | 2.51E-05* |
| Endotoxin (NS) | 0 (0.00) | 32 (1.80) | 2.98E-03* |
| Type II secretion system (NS) | 0 (0.00) | 22 (1.24) | 4.24E-02* |
| Regulation (R) | 2 (0.39) | 32 (1.80) | 1.01E-01 |
| Intracellular survival (NS) | 0 (0.00) | 12 (0.68) | 2.93E-01 |
| Adherence (O) | 78 (15.09) | 319 (17.99) | 4.06E-01 |
| Cell wall (NS) | 0 (0.00) | 11 (0.62) | 4.06E-01 |
| Secretion system (other)^f^ (NS) | 32 (6.19) | 141 (7.95) | 4.97E-01 |
| Cellular metabolism (D) | 0 (0.00) | 9 (0.51) | 4.97E-01 |
| Enzyme (O) | 0 (0.00) | 8 (0.45) | 4.97E-01 |
| Invasion (O) | 2 (0.39) | 18 (1.02) | 5.87E-01 |
| Exoenzyme (NS) | 5 (0.97) | 30 (1.69) | 6.19E-01 |
| Immune evasion (D) | 1 (0.19) | 10 (0.56) | 8.33E-01 |
| Biofilm formation (D) | 0 (0.00) | 4 (0.23) | 9.46E-01 |
| Molecular mimicry (NS) | 0 (0.00) | 4 (0.23) | 9.46E-01 |
| IgA1 Protease (D) | 0 (0.00) | 3 (0.17) | 1.00E+00 |
| Intracellular growth (NS) | 0 (0.00) | 3 (0.17) | 1.00E+00 |
| Magnesium uptake (NS) | 0 (0.00) | 3 (0.17) | 1.00E+00 |
| Serum resistance (D) | 0 (0.00) | 3 (0.17) | 1.00E+00 |
| Biosurfactant (NS) | 0 (0.00) | 2 (0.11) | 1.00E+00 |
| Complement protease (D) | 0 (0.00) | 2 (0.11) | 1.00E+00 |
| Pigment (O) | 0 (0.00) | 2 (0.11) | 1.00E+00 |
| Secreted proteins (O) | 0 (0.00) | 2 (0.11) | 1.00E+00 |
| Bile resistance (D) | 0 (0.00) | 1 (0.06) | 1.00E+00 |
| Complement resistance (D) | 0 (0.00) | 1 (0.06) | 1.00E+00 |
| Heat-shock protein (NS) | 0 (0.00) | 1 (0.06) | 1.00E+00 |
| Manganese uptake (NS) | 0 (0.00) | 1 (0.06) | 1.00E+00 |
| Nutrient acquisition (NS) | 0 (0.00) | 1 (0.06) | 1.00E+00 |
| Peptidase (D) | 0 (0.00) | 1 (0.06) | 1.00E+00 |
| Resistance to antimicrobial peptides (D) | 0 (0.00) | 1 (0.06) | 1.00E+00 |

^a^VFs are defined as those genes curated as being VFs according to the VFDB. VFs are also categorized, according to the VFDB, as O = Offensive; D = Defensive; NS= Nonspecific; R = Regulation; NA = Not Available

^b^Based on the percentage of pathogen-associated or “Common” VFs in a given functional category

^c^Fisher’s Exact test followed by the Benjamini-Hochberg multiple testing correction. Asterisks indicate statistical significance (p-value < 0.05).

^d^Includes Type III secretion system genes and Type III translocated proteins.

^e^Includes Type IV secretion system genes and Type IV secretory proteins.

^f^Includes Secretion system genes not classified as Type II, III or IV secretion system
