# Supplementary material for: The Association of Virulence Factors with Genomic Islands
Source: PLoS One. 2009 Dec 1;4(12):e8094. doi: 10.1371/journal.pone.0008094 (PMC2779486; doi:10.1371/journal.pone.0008094)
Supplement: Table S2 — Analysis of VFDB virulence factors in a set of GIs derived from whole-genome comparisons by Vernikos and Parkhill (2008). (0.06 MB DOC) [file pone.0008094.s004.doc]

## Table S2 - Association of VFDB virulence factors (VFs) with genomic islands (GIs) defined by Vernikos *et al.* (2008).

| **Organism** | **Accession** | **#GIs** | **Total GI Length** | **# Genes in GIs** | **# VFs** | **# VFs in GIs** | **# nonVFs in GIs** | **# VFs in non-GIs** | **# nonVFs in non-GIs** | **Fisher**  ***p*-value** |
| --- | --- | --- | --- | --- | --- | --- | --- | --- | --- | --- |
| *Salmonella typhi* | NC_003198 | 123 | 1010126 | 990 | 166 | 111 | 879 | 55 | 3350 | 1.29E-35 |
| *Salmonella typhi Ty2* | NC_004631 | 206 | 100103 | 503 | 166 | 66 | 437 | 100 | 3715 | 1.65E-21 |
| *Salmonella typhimurium LT2* | NC_003197 | 208 | 737165 | 778 | 156 | 83 | 695 | 73 | 3574 | 6.79E-25 |
| *Staphylococcus aureus aureus MRSA252* | NC_002952 | 182 | 472927 | 341 | 73 | 23 | 318 | 50 | 2265 | 1.93E-05 |
| *Staphylococcus aureus MW2* | NC_003923 | 173 | 379862 | 288 | 54 | 28 | 260 | 26 | 2318 | 4.94E-14 |
| *Staphylococcus aureus Mu50* | NC_002758 | 241 | 407594 | 369 | 85 | 36 | 333 | 49 | 2279 | 3.56E-11 |
| *Staphylococcus aureus N315* | NC_002745 | 262 | 400179 | 269 | 86 | 38 | 231 | 48 | 2271 | 1.57E-16 |
| *Staphylococcus aureus RF122* | NC_007622 | 136 | 189531 | 258 | 79 | 34 | 224 | 45 | 2206 | 1.30E-14 |
| *Staphylococcus aureus USA300* | NC_007793 | 286 | 382823 | 255 | 78 | 34 | 221 | 44 | 2261 | 3.08E-15 |
| *Staphylococcus epidermidis ATCC 12228* | NC_004461 | 11 | 112386 | 118 | 13 | 1 | 117 | 12 | 2289 | 0.48 |
| *Staphylococcus epidermidis RP62A* | NC_002976 | 23 | 175719 | 211 | 18 | 0 | 211 | 18 | 2265 | 1 |
| *Staphylococcus haemolyticus* | NC_007168 | 10 | 208810 | 254 | 17 | 0 | 254 | 17 | 2405 | 1 |
| *Staphylococcus saprophyticus* | NC_007350 | 12 | 52778 | 36 | 19 | 0 | 36 | 19 | 2391 | 1 |
| *Streptococcus agalactiae A909* | NC_007432 | 36 | 137344 | 158 | 50 | 1 | 157 | 49 | 1789 | 0.98 |
| *Streptococcus agalactiae NEM316* | NC_004368 | 16 | 253544 | 261 | 54 | 1 | 260 | 53 | 1780 | 1 |
| *Streptococcus pneumoniae R6* | NC_003098 | 13 | 32398 | 38 | 25 | 0 | 38 | 25 | 1980 | 1 |
| *Streptococcus pneumoniae TIGR4* | NC_003028 | 23 | 137477 | 132 | 47 | 23 | 109 | 24 | 1949 | 1.50E-16 |
| *Streptococcus pyogenes MGAS10750* | NC_008024 | 79 | 61502 | 164 | 38 | 4 | 160 | 34 | 1781 | 0.39 |
| *Streptococcus pyogenes MGAS2096* | NC_008023 | 23 | 171600 | 166 | 38 | 2 | 164 | 36 | 1696 | 0.86 |
| *Streptococcus pyogenes MGAS9429* | NC_008021 | 61 | 53063 | 109 | 42 | 4 | 105 | 38 | 1730 | 0.22 |
| *Streptococcus thermophilus CNRZ1066* | NC_006449 | 29 | 71366 | 87 | 30 | 19 | 68 | 11 | 1817 | 1.51E-19 |
| *Streptococcus thermophilus LMG 18311* | NC_006448 | 5 | 25768 | 27 | 38 | 2 | 25 | 36 | 1826 | 0.10 |
| **TOTALS** |  | **2158** | **5574065** | **5812** | **1372** | **510** | **5302** | **862** | **49937** | **9.48E-160** |
