# Supplementary material for: The Association of Virulence Factors with Genomic Islands
Source: PLoS One. 2009 Dec 1;4(12):e8094. doi: 10.1371/journal.pone.0008094 (PMC2779486; doi:10.1371/journal.pone.0008094)
Supplement: Table S3 — Enrichment of Swiss-Prot-derived virulence proteins in GIs. (0.03 MB DOC) [file pone.0008094.s005.doc]

## Table S3 - Association of Swiss-Prot virulence factors (SPVFs) with genomic islands (GIs).

| **GI Identification Method** | **# SPVFs in GIs** | **# genes in GIs** | **% SPVFs in GIs** | **# SPVFs in non-GIs** | **# genes in non-GIs** | **% SPVFs in non-GIs** | **Fisher *p*-value** |
| --- | --- | --- | --- | --- | --- | --- | --- |
| IslandPath-DINUC^a^ | 103 | 15650 | 0.66 | 7 | 21611 | 0.03 | 9.21E-31 |
| IslandPath-DIMOB^b^ | 10 | 5859 | 0.17 | 7 | 21611 | 0.03 | 8.44E-04 |
| SIGI-HMM^c^ | 17 | 3616 | 0.47 | 7 | 21611 | 0.03 | 5.54E-10 |

^a^GIs are defined as 8 or more consecutive ORFs with dinucleotide bias as predicted with IslandPath-DINUC.

^b^GIs are defined as 8 or more consecutive ORFs with dinucleotide bias plus presence of 1 or more mobility genes within the region as predicted with IslandPath-DIMOB.

^c^GIs are defined based on codon usage (removing regions like ribosomal operons) as predicted with SIGI-HMM. See text regarding the complementarity of the IslandPath-DIMOB and SIGI-HMM methods.
