# Supplementary material for: The Association of Virulence Factors with Genomic Islands
Source: PLoS One. 2009 Dec 1;4(12):e8094. doi: 10.1371/journal.pone.0008094 (PMC2779486; doi:10.1371/journal.pone.0008094)
Supplement: Text S1 — Supplemental methods. (0.03 MB DOC) [file pone.0008094.s006.doc]

## Methods

**Association of VFDB virulence factors (VFs) with genomic islands (GIs) defined by Vernikos & Parkhill (2008)**

We performed an analysis of virulence factors in the set of GIs defined by Vernikos & Parkhill (2008). This set of GIs was derived from whole-genome comparisons to identify genomic regions with limited phylogenetic distribution consistent with recent acquisition. GI coordinates were obtained from the supplemental research data associated with this paper, using Supp. Table 1 to obtain genomic coordinates for GIs for each genome. This information was manually cross-referenced with Supp. Figure S11 to determine the phylogenetic distribution of each GI, and the corresponding regions were identified in each of the genomes using a BLASTN search using an e-value cutoff of 1E-100. The number of virulence factors in each GI were tabulated and compared to the number of virulence factors outside of GIs. Additionally, we determined the total number of genes in and outside of GIs.

**Association of virulence proteins from Swiss-Prot with GIs**

We performed an analysis of virulence factors in GIs using an independently extracted set of virulence factors from Swiss-Prot. Specifically, we extracted all proteins from Swiss-Prot that are annotated with “virulence” as the “Biological Process”. We then filtered the set to retain only those occurring in complete proteomes from Bacteria. This gave us a set of 1029 proteins, all with “reviewed” status, which we called “Swiss-Prot virulence factors” or SPVFs. We then determined the number of SPVFs in genomic islands predicted by IslandPath-DINUC, IslandPath-DIMOB, and SIGI-HMM, and compared it to the number of SPVFs in a dataset of genomic regions that were not likely to contain GIs, i.e. large genomic regions that were conserved in several genomes. This negative set was used in an evaluation of genomic island predictors published by Langille et al. (2008) in BMC Bioinformatics.
